# An improved Tet-on system in microRNA overexpression and CRISPR/Cas9-mediated gene editing

**DOI:** 10.1186/s40104-019-0354-5

**Published:** 2019-06-10

**Authors:** Kang Kang, Lian Huang, Qing Li, Xiaoyun Liao, Quanjin Dang, Yi Yang, Jun Luo, Yan Zeng, Li Li, Deming Gou

**Affiliations:** 1 Department of Biochemistry and Molecular Biology, Carson International Cancer Center, Shenzhen University Health Sciences Center, Shenzhen, Guangdong 518060 People’s Republic of China; 2 Shaanxi Key Laboratory of Molecular Biology for Agriculture, College of Animal Science and Technology, Northwest A&F University, Yangling, Shaanxi 712100 People’s Republic of China; 3 Shenzhen Key Laboratory of Microbial Genetic Engineering, College of Life Sciences and Oceanography, Shenzhen University, Xueyuan Ave 1066, Shenzhen, Guangdong 518060 People’s Republic of China

**Keywords:** CRISPR/Cas9, Doxycycline, microRNA, NFAT5, Tetracycline

## Abstract

**Background:**

Tetracycline (Tet)-regulated expression system has become a widely applied tool to control gene activity. This study aimed to improve the Tet-on system with superior regulatory characteristics.

**Results:**

By comprehensively comparing factors of transactivators, Tet-responsive elements (TREs), orientations of induced expression cassette, and promoters controlling the transactivator, we developed an optimal Tet-on system with enhanced inducible efficiency and lower leakiness. With the system, we successfully performed effective inducible and reversible expression of microRNA, and presented a more precise and easily reproducible fine-tuning for confirming the target of a miRNA. Finally, the system was applied in CRISPR/Cas9-mediated knockout of nuclear factor of activated T cells-5 (*NFAT5*), a protective transcription factor in cellular osmoregulation.

**Conclusions:**

This study established an improved Tet-on system for powerful and stringent gene regulation in functional genetic studies.

**Electronic supplementary material:**

The online version of this article (10.1186/s40104-019-0354-5) contains supplementary material, which is available to authorized users.

## Background

Tet-responsive expression system is a promising tool for gene functional analysis and gene therapy. The system can be divided into Tet-off and Tet-on types, based on whether gene expression is allowed in the absence or presence of Tet, respectively. The Tet-off system includes two different units: one is TREs consisting of multiple Tet operators (TetOs) of *E. coli* Tn10 upstream of a minimal RNA polymerase II promoter; the other is a Tet-regulated transactivator (tTA), a fusion protein of Tet repressor (TetR) and a transcriptional transactivator VP16 of herpes simplex virus [1]. The Tet-off system is usually used in induced gene expression lasting for a long period such as animal-based *in vivo* experiments. However, sustained presence of Tet or its derivative doxycycline (Dox) is required to maintain the un-induced state, which might cause side effect on the physiology of mammals. In the Tet-on system, tTA is replaced by a mutant reverse tTA (rtTA), which is capable of binding to TREs and activating gene expression only in the presence of Dox [2].

The original version of rtTA has several limitations, such as the requirement of high concentrations of Dox for full activation, and the existence of high background activity or leakiness. Urlinger and colleagues developed new rtTAs by random mutagenesis and codon optimization [3–5]. They successfully identified novel types of rtTA, rtTA2^S^-M2, and rtTA2^S^-S2, with higher sensitivity to the inducer and lower basal activity in the absence of Dox. Moreover, by using a viral evolution system, Das and his colleages obtained some valuable mutated forms of rtTA, which displayed enhanced expression activity and Dox-sensitivity, as well as reduced background [6–8]. Apart from improving the feature of transactivator, efforts were also put into optimizing the *cis* elements to which transactivators bind. By fine-tuning the TATA box flanking sequence in the minimal promoter, several promoters with less leakiness and higher inducibility were acquired [9, 10].

Currently, there are two commonly used Tet-on inducible vectors, pLVX-TetOne-Puro (Clontech, Mountain View, CA, USA) [11, 12] and pTRIPZ (Thermo Fisher Scientific, Huntsville, AL, USA) [13, 14], which all belong to the so-called third generation of Tet systems. pLVX-TetOne-Puro uses TetON3G as a transactivator and TRE3Gs as the TREs, while pTRIPZ uses rtTA3 and TetO6 correspondingly. Compared with the original rtTA, both TetON3G and rtTA3 contain valuable amino acid mutations, for example, E19G, A56P, F86Y, and A209T. Mutation of E19G and A56P not only confers reverse tTA feature, but also allows minute background activity [4]. The combination of A209T and F86Y, and even F86Y individually, significantly increases the transcriptional activity of rtTA [8].

To further improve the Tet-on inducible system, we systematically compared different transactivators, TREs, orientations of expression cassettes and multiple promoters controlling the transactivator. By combining a series of advantageous factors, a tighter and more efficient Tet-on system was developed. With the optimized Tet-on system, we achieved excellent inducible and reversible expression of microRNAs (miRNAs). We further demonstrated a more precise, easily reproducible and cost-effective fine-tuning confirming the target gene of a miRNA. Last, the system was used with the CRISPR/Cas9 technique for the genetic perturbation of *NFAT5*, a transcription factor involved in cellular adaptation to hypertonic stress.

## Methods

### Plasmid construction

The plasmid pLVX-Puro (Clontech) was used as a backbone vector and sequentially subjected to *Tth*111I digestion, blunting, *Cla*I restriction, and recovery of the vector backbone. An artificially synthesized MCS-P_PGK_-TetON3G-P2A-Puro fragment was PCR-amplified with Pfu DNA Polymerase (Promega, Madison, WI, USA) and excised with *Cla*I, followed by ligation to the above recovered pLVX-Puro, generating pLVX-Tet2A-Puro. Then, the coding sequence of firefly luciferase (Luc) PCR-amplified from plasmid pmirGLO (Promega) was excised with *Not*I and *Mlu*I and ligated to the equally restricted pLVX-Tet2A-Puro to become pLVX-Luc-Tet2A-Puro. Next, artificially synthesized fragments of TRE3Gs, TRE3Gp, and TetO6 were excised with *Xba*I and *Not*I, and ligated to the equally cleaved pLVX-Luc-Tet2A-Puro, yielding the plasmids p1, p2, and p3. To replace TetON3G, the fragment of reverse tetracycline-transactivator 3 (rtTA3) PCR-amplified from plasmid pTRIPZ was digested with *Bst*BI and *Sma*I, and ligated to equally restricted p1, p2, and p3, producing plasmids p4, p5, and p6. To develop plasmids with an induced expression cassette in the same orientation, TRE3Gs-Luc, TRE3Gp-Luc, and TetO6-Luc fragments were excised from p1, p2, and p3 with *Bsm*BI, respectively, and ligated to *Mlu*I/*Xba*I-restricted p1 or p4, creating plasmids p7, p8, p9, p10, p11, and p12. Promoters P_CMV_, P_EF1α_, P_SV40_, and P_Ubc_ were PCR-amplified from plasmids pIRESneo-FLAG-HA-Ago2 (Addgene, Cambridge, MA, USA), pCDH-CMV-MCS-EF1-copGFP (SBI, Mountain View, CA, USA), pmirGLO, and pTRIPZ, respectively, and ligated to p2 between *Xba*I and *Bst*BI to replace *P*_PGK_, generating plasmids p13, p14, p15, and p16.

To inducibly express microRNA, the primary sequence of three miRNAs (hsa-miR-210, hsa-miR-21, and hsa-miR-26a) and the coding sequence of enhanced green fluorescent protein (EGFP) were sequentially inserted into p2 via *Mlu*I/*Cla*I and *Not*I/*Mlu*I, respectively. A plasmid with an *EGFP* fragment but lacking any primary miRNA sequence was used as a negative control (NC). To construct a vector inducibly expressing Cas9, a Cas9-FLAG-P2A-Puro fragment PCR-amplified from LentiCRISPR v2 (Addgene) was excised with *Not*I and *Mlu*I, and ligated to equally restricted p2; meanwhile, the *EGFP* coding sequence was applied to take the place of the *puro* fragment of p2 between *Bam*HI and *Spe*I, together generating a plasmid pL-Cas9. The artificially synthesized DNA sequence and primers used in this study are presented in Additional file 3.

### Cell culture

The HEK293A, HEK293T, HeLa, A549 and mIMCD3 cells were purchased from American Type Culture Collection (ATCC, Manassas, VA, USA). Cells were cultured in DMEM with 10% FBS in a humidified incubator with 5% CO_2_ at 37 °C. Primary human pulmonary arterial smooth muscle cells (hPASMCs) were purchased from Lonza (Walkersville, MD) and cultured in SmGM-2 smooth muscle growth media consisting with smooth muscle basal medium, 5% FBS, 0.5 ng/mL human recombinant epidermal growth factor, 2 ng/mL human recombinant fibroblast growth factor, 5 μg/mL insulin, and 50 μg/mL gentamicin.

### Lentiviral packaging, transduction, and inducibility

The lentivirus particles were prepared in HEK293T cells by transfection of the following three plasmids at a ratio of 2:1:5 - (i) psPAX2 (Addgene), (ii) pCMV-VSV-G (Addgene), and (iii) a lentivirus vector. Briefly, 2 × 10^6^ HEK293T cells were seeded in 10-cm culture dishes. After 12 h of incubation, cells were transfected with packaging plasmids (7.5 μg of a mixture of psPAX2 and pCMV-VSV-G) and lentivirus vector (12.5 μg) using PEI reagents. The culture supernatants were harvested 48 h after transfection and used for cell infection in the presence of 5 μg/mL polybrene. At 48 h after infection, the infected HEK293A cells were selected in 2 μg/mL puromycin for 5–7 d. The puromycin-resistant cells were expanded and cultured in the presence of different concentrations of Dox for inducible analysis.

### Quantitative RT-PCR (qRT-PCR)

Total RNA was extracted with RNAiso Plus (TaKaRa, Dalian, China). For miRNA evaluation, the mature miRNAs were detected using the S-Poly(T) plus method [15, 16]. For the mRNA assay, the SYBR Green method was used with oligo (dT) plus random primers to initiate cDNA synthesis [17]. The miRNA and mRNA expression levels were normalized to SNORD44 and β-actin, respectively, and calculated using the 2^-ΔΔCt^ method. Primers used in reverse transcription and qPCR are listed in Additional file 3.

### Luciferase activity assay

For transient transfection, cells were seeded in 48-well plates. After reaching 80% confluence, cells were transfected with 400 ng of luciferase inducible plasmid and 40 ng of phRL-TK using polyethylenimine (PEI) reagents. Six hours after transfection, cells were changed to fresh medium in the presence of different concentrations of Dox and incubated for 48 h. Cells were harvested for luciferase activity assay and measured with a Lumat LB9508 luminometer (Berthold, Bad Wildbad, Germany). Firefly activity was normalized to *Renilla* luciferase activity. Fold induction was defined as the ratio between the induced expression level with Dox and the background expression level without Dox.

To perform luciferase activity assay by lentivirus integration, hPASMC and A549 cells were infected with p2, p13, p14, p15 and p16 lentivirus, respectively, and then selected with puromycin. The stable cell lines were induced with 1 μg/mL Dox for 48 h. Then, equal numbers of stable cells were assayed for luciferase activity. Firefly luciferase values were normalized to the copy number of luciferase integrated into the genome of each stably cell line, which was determined by quantitative PCR.

### Western blotting

Cells were lysed with cold RIPA buffer (50 mmol/L Tris-HCl, pH 7.5, 150 mmol/L NaCl, 1% NP-40, 0.25% sodium deoxycholate, and 1 mmol/L EDTA) supplemented with protease inhibitor cocktail (Roche, Mannheim, Germany) and quantified with the bicinchoninic acid protein assay kit (Thermo Fisher Scientific). Equal amounts of protein (~ 30 μg) were subjected to SDS-PAGE and transferred to PVDF membranes. After blocking with 5% skimmed milk in TBST (20 mmol/L Tris-HCl, pH 7.6, 150 mmol/L NaCl, and 0.1% Tween 20), membranes were incubated with primary antibodies overnight at 4 °C and then with HRP-conjugated secondary antibodies. The protein bands were visualized with the chemiluminescent detection module (Pierce Biotechnology, Rockford, IL, USA) and images were taken with the Tanon-5200 imaging system (Tanon, Shanghai, China). The following primary antibodies were used: PDCD4 (Santa Cruz Biotechnology, Santa Cruz, CA, USA, SC-13054, 1:1,000), *NFAT5* (Santa Cruz Biotechnology, SC-13035, 1:200), FLAG (GenScript, Piscataway, NJ, USA, A00013, 1:1,000), β-actin (Proteintech, Wuhan, Hubei, China, 66009–1-Ig, 1:10,000;), and β-tubulin (Proteintech, 10094-I-AP, 1:5,000).

### Stable Cas9 cell line

FLAG-Cas9 Lentivirus was produced as described above. After lentiviral infection for 24 h, cells were changed to fresh medium with 1 μg/mL Dox for 48 h. Subsequently, the infected HEK293A cells were selected in 2 μg/mL puromycin for 5–7 d. Next, single HEK293A cells were picked up and cultured in 96-well plates. After ~ 7 d, the cell colonies were subcultured sequentially in 24- and 6-well plates with 1 μg/mL puromycin for another 10 d. Subsequently, a fraction of selected cells were subjected to western blotting analysis, and the rest were frozen for future use.

### sgRNA *in vitro* production, gene targeting, and phenotypic analysis

Four sgRNAs targeting the human *NFAT5* gene were designed using Broad Institute CRISPRko software [18] (Additional file 3). The sgRNA targeting red fluorescence protein (*RFP*) was used as a NC. sgRNAs were *in vitro* generated according to the instructions of the EnGen sgRNA Synthesis Kit (NEB, Beverly, MA, USA). Briefly, to add the T7 promoter to the sgRNA coding sequences, LentiCRISPR v2 (Addgene) as a template was PCR-amplified using CRISPR-specific forward primers and a universal reverse primer. The PCR products were used as templates for *in vitro* transcription with the TranscriptAid T7 High Yield Transcription Kit (Thermo Fisher Scientific). The resultant sgRNAs were subjected to alkaline phosphatase treatment, phenol-chloroform extraction, dissolved in RNase-free water and stored at − 80 °C until use.

The Cas9 stable cell line was treated with Dox (1 μg/mL) for 2 d before and during transfection. For transfection, cells were seeded in 6-well plates and transfected with each of the four sgRNAs or a mixture of them at a final concentration of 50 nmol/L using METAFECTENE SI^+^ reagent (Biontex Laboratories GmbH, Munich, Germany). At 24 h after transfection, cells were transferred to fresh medium with 1 μg/mL Dox and 1 μg/mL puromycin for 2 d. Next, cell colonies were picked up and subcultured sequentially in 96-, 24-, and 6-well plates with 1 μg/mL puromycin. Subsequently, a fraction of selected cells were subjected to phenotypic analysis, and the rest were frozen for future use.

For phenotypic analysis, sgRNA-transfected cell lines were cultured in isotonic (300 mOsm/kg) or hypertonic (550 mOsm/kg) media for 8 and 24 h. Cells cultured for 8 h were harvested for *NFAT5* protein analysis by western blotting and TauT/SMIT expression assay by qRT-PCR. Cells cultured for 24 h were subjected to a cell viability assay using the CellTiter 96 Aqueous One Solution Cell Proliferation Assay (Promega) [19, 20].

### T7 endonuclease I assay

Genomic DNA was isolated from cells transfected with sgRNA targeting *RFP* (NC) and *NFAT5*. Target regions were PCR-amplified from genomic DNA with specific primers (Additional file 3). Then, 250 ng of purified PCR product were denatured at 95 °C for 10 min and re-annealed at − 2 °C per second temperature ramp to 85 °C, followed by a − 1 °C per second ramp to 25 °C. The mismatched DNA were then digested with 2 units of T7 endonuclease I (T7E1) (GeneCopoeia, Guangzhou, China) for 1 h at 37 °C and separated by 2% agarose gel electrophoresis. Digestion efficiency was calculated by measuring band intensities with ImageJ (NIH version 1.6).

### Statistical analysis

Each experiment was repeated at least three times. Data are presented as mean ± standard deviation (SD). Statistical analysis of the data was performed using a two-tailed Student’s *t*-test with GraphPad Prism 5 (GraphPad, San Diego, CA, USA). A *P*-value of < 0.05 was considered statistically significant.

## Results

### Optimal combination of TREs and transactivator

**Fig. 1:**
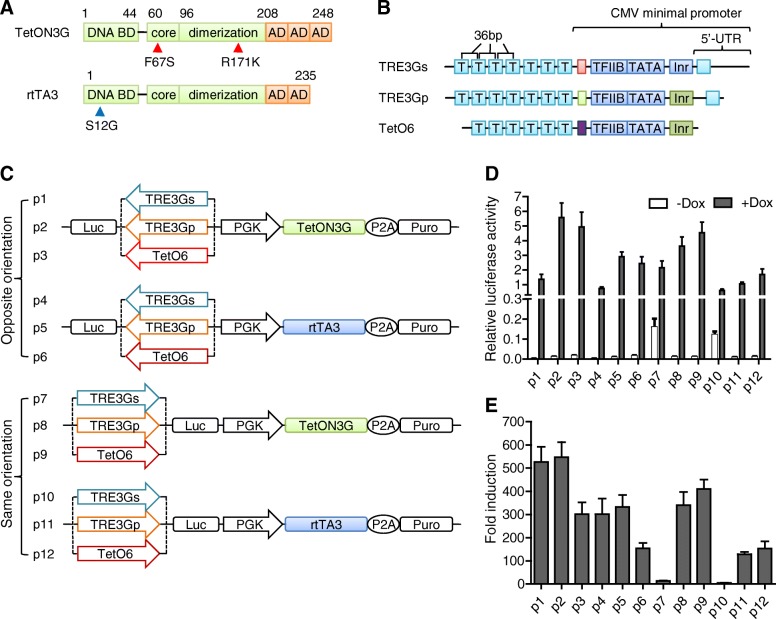
Comparison of the induced efficacies of twelve Tet-on plasmids. **a** Comparison of two transactivators. Both TetON3G and rtTA3 were fusions of TetR and several VP16-derived minimal ADs. TetR is composed of a DNA binding domain (BD) and a core domain following a dimerization surface. The F67S, R171K (red arrow), and S12G (blue arrow) mutations of amino acids are indicated. **b** Comparison of three TREs: TRE3Gs, TRE3Gp, and TetO6. These TREs contain seven (TRE3Gs and TRE3Gp) or six (TetO6) TetOs with a 36-bp center-to-center distance. Moreover, they have TFIIB and a TATA box, as well as a predicted initiator (Inr), but differ in the length and composition of the 5′-UTR sequence. T: TetO6. **c** Schematic representation of different combinations of two transactivators, three TREs, and two orientations (the same and the opposite) of inducible expression cassette. **d** The induced efficiency of 12 Tet-on plasmids (p1-p12). HEK293A cells were transfected with each of the 12 plasmids with firefly luciferase as a reporter gene. Cells were grown in the absence or presence of 1 μg/mL Dox. After 48 h, cells were harvested for a luciferase activity assay. Firefly luciferase activity was normalized to *Renilla* luciferase activity. **e** The fold induction of the 12 plasmids. Fold induction was defined as the ratio between the induced expression level with Dox and the background expression level without Dox

To improve the Tet-on inducible system, we evaluated the effects of two transactivators (rtTA3 and TetON3G), three TREs (TRE3Gs, TRE3Gp and TetO6), and two orientations of the inducible expression cassette, using a total of 12 inducible plasmids p1-p12 (Fig. 1a-c, Additional file 3). Luc was used as a reporter gene to assess the background activity and inducible efficiency. As Fig. 1d indicates, TetON3G had higher transcriptional activity than rtTA3 (for example, p1-p3 vs. p4-p6). Among the three TREs, both TRE3Gp and TetO6 surpassed TRE3Gs (for example, p2 and p3 vs. p1). With regard to orientation, the induced expression cassette in the opposite orientation (p1-p6) displayed higher Luc activity than those in the same orientation (p7-p12). TRE3Gp was dominant in this regard in the opposite orientation, while TetO6 displayed superiority in the same one. Meanwhile, the TRE3Gs expression cassette in the same orientation showed a remarkable background activity, indicating that these TREs, particularly TRE3Gs, have an orientation-dependent effect.

We further used fold induction (the ratio between the induced expression and the uninduced background activity) to evaluate these constructs. The plasmid p2, with a combination of TetON3G and TRE3Gp in an opposite orientation, had a maximal fold induction of ~ 550 (Fig. 1e), being the optimal inducible system among them. We hence applied p2 in the subsequent experiments.

### Comparison of promoters controlling transactivator expression

**Fig. 2:**
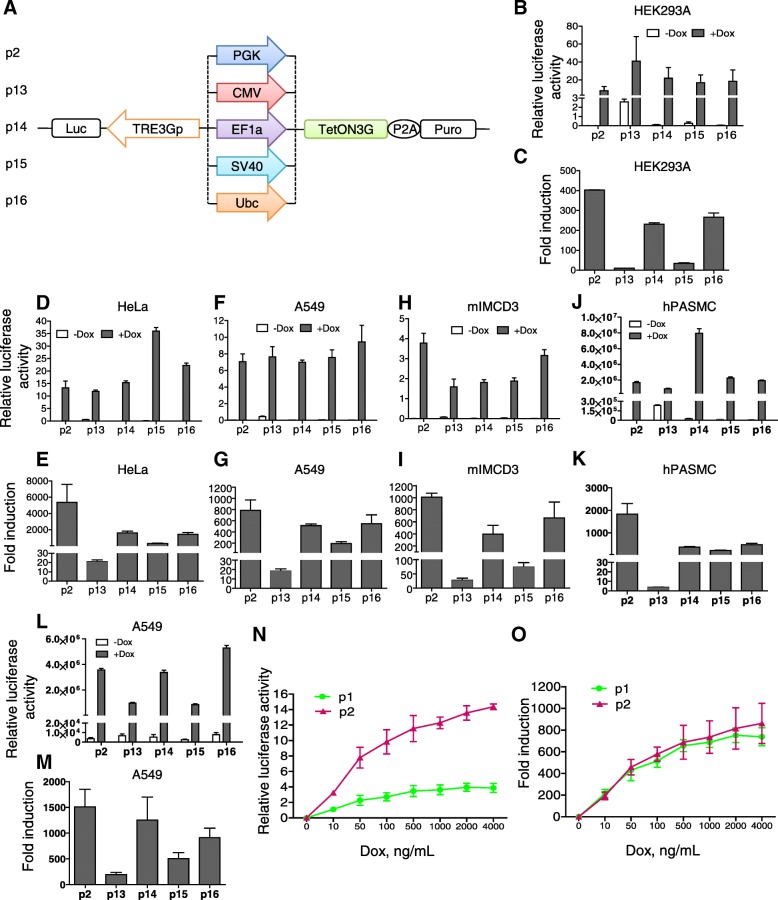
Comparison of promoters controlling transactivator expression. **a** Schematic representation of five plasmids with different promoters, namely, P_PGK_, P_CMV_, P_EF1α_, P_SV40_, and P_Ubc_, for controlling the expression of the transactivator. B~I: The inducible efficiency of five plasmids (p2, p13-p16). HEK293A (**b**), HeLa (**d**), A549 (**f**) and mIMCD3 (**h**) cells were transfected with each of the five plasmids with firefly luciferase as a reporter gene, respectively. Cells were grown in the absence or presence of Dox. Then, cells were harvested for luciferase activity assay. Firefly luciferase activity was normalized to *Renilla* luciferase activity. The fold induction of luciferase activity of these plasmids in HEK293A (**c**), HeLa (**e**), A549 (**g**) and mIMCD3 (**i**) cells were calculated as the ratio between the induced expression level with Dox and the background expression level without Dox. **j**-**m** The inducible efficiency of the five plasmids assessed by lentiviral infection. Cells of hPASMC (**j**) and A549 (**l**) were infected with corresponding lentivirus. The stable cell lines were induced with Dox. Then, equal numbers of stable cells were assayed for luciferase activity. Firefly luciferase values were normalized to the copy number of luciferase integrated into the genome of each stably cell line, which was determined by quantitative PCR. The fold induction of luciferase activity in hPASMC (**k**) and A549 (**m**) were also calculated. **n** The Dox sensitivity of the inducible system. HEK293A cells transfected with plasmids p1 and p2 were grown under different concentrations of Dox (0, 10, 50, 100, 500, 1,000, 2,000, and 4,000 ng/mL), and subjected to a luciferase activity assay 48 h after transfection. **o** The fold induction of luciferase activity between plasmids p1 and p2

The promoter controlling transactivator expression is also an important factor affecting the inducible efficiency. Five promoters, P_PGK_, P_CMV_, P_EF1α_, P_SV40_, and P_Ubc_, driving the TetON3G expression were quantified and compared in Luc induction in HEK293A cells by plasmid transfection (p2, p13-p16; Fig. 2a). These promoters displayed different activities based on the luciferase assay (Fig. 2b). Among them, P_CMV_ is the strongest, while P_PGK_ is the weakest, and the other three are moderate. However, P_CMV_ displayed strong leaky activity in the absence of Dox. P_PGK_ is the best promoter for the transactivator TetON3G in terms of fold induction (Fig. 2c). To test whether such a superiority of P_PGK_ is cell-specific, we also evaluated these promoters in other cell lines including two human cancer cell lines, HeLa cell and A549 cell, and a mouse cell line, mIMCD3. The results showed that P_PGK_ is consistently the best promoter among the tested in terms of fold induction, while P_CMV_ is the leakiest in all cell lines tested (Fig. 2d-i).

To assess the influence of transient transfection of plasmid DNA on luciferase activity, meanwhile enlarge its application range of our Tet-on system, we also compared promoters in controlling the transactivators by lentiviral integration. The primary human pulmonary arterial smooth muscle cells (hPASMCs), as well as A549 cells were sequentially infected by p2, p13, p14, p15 and p16 lentivirus, respectively, selected by puromycin, and induced with Dox. The results showed that luciferase values obtained through lentiviral integration was similar to those via transient transfection, and that P_PGK_ was still superior to other four promoters tested in both hPASMC and A549 cells (Fig. 2j-m).

To evaluate the inducible sensitivity to Dox, we tested p1 and p2 in Luc expression under different Dox levels. As shown in Fig. 2n, a notable luciferase reading was observed when the concentration of Dox was as low as 10 ng/mL, and the reading gradually elevated with increasing amount of Dox. Overall, the plasmid p2 surpassed p1 under each Dox concentration in terms of both absolute induction and fold induction (Fig. 2n and o). Moreover, p2 had a broader dynamic range of induced expression than p1, suggesting that p2 is more sensitive to Dox than p1.

### Precisely regulating expression of miRNA and its target

**Fig. 3:**
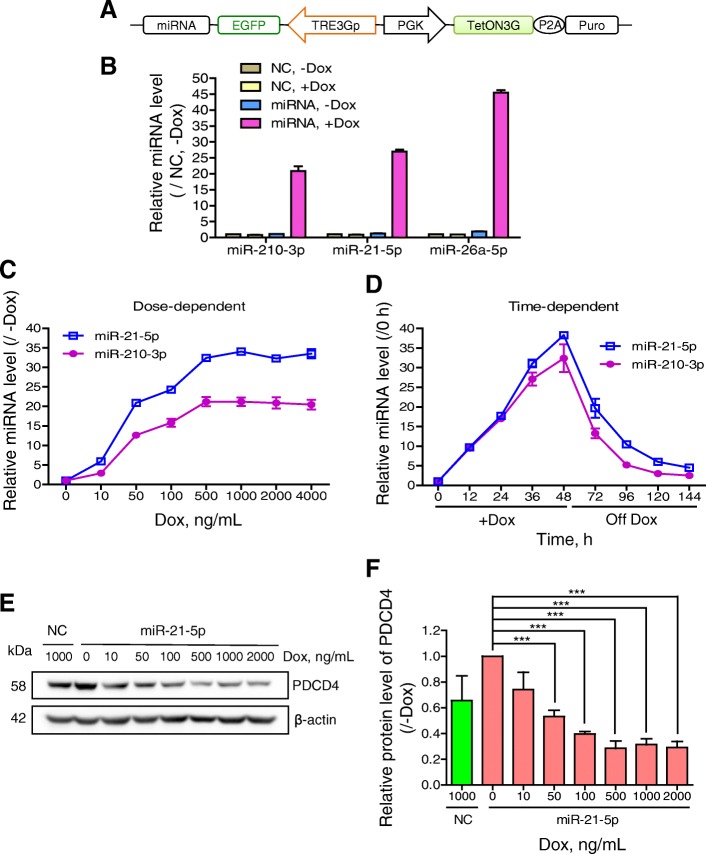
Precise regulation of miRNA expression and its target. **a** Schematic representation of p2-based inducible miRNA expression plasmid. The EGFP and the downstream primary miRNA sequence were positioned under the control of the TREs. **b** Inducible miRNA expression. HEK293A cells were infected with lentiviruses carrying the primary sequences of hsa-miR-210, hsa-miR-21, and hsa-miR-26a, and cultured in the absence or presence of 1 μg/mL Dox for 48 h. Cells were harvested and subjected to measurement of the miRNA level by qRT-PCR. Lentivirus without any primary miRNA sequence was used as a NC. The level of each miRNA was calculated as the fold change relative to that in NC without Dox. **c** The dose-dependent effect of miRNA inducibility. miR-210-3p and miR-21-5p were induced under different concentrations of Dox (0, 10, 50, 100, 500, 1,000, 2,000, and 4,000 ng/mL) for 48 h and subjected to measurement of the miRNA level. The level of each miRNA was calculated as the fold change relative to that without Dox. **d** The time-dependent and reversible effect of miRNA inducibility. miR-210-3p and miR-21-5p were induced with 1 μg/mL Dox for 0, 12, 24, 36, and 48 h. Subsequently, cells that had undergone 48-h induction were subcultured in fresh medium without Dox for another 24, 48, 72, and 96 h, and harvested for miRNA detection. **e** The PDCD4 protein levels of cells inducibly expressing miR-21-5p under different Dox concentrations were assayed by western blotting. Beta-actin served as a loading control. **f** The PDCD4 protein levels were quantified. The data are expressed relative to that without Dox, and are presented as mean ± SD (*n* = 3). ***P < 0.001

miRNA can post-transcriptionally regulate gene expression [21, 22]. To test the Tet-on system in inducible miRNA expression, the primary sequences of three miRNAs, hsa-miR-210, hsa-miR-21, and hsa-miR-26a, were inserted downstream of the TREs (Fig. 3a). Mature miRNAs were detected after Dox induction. In the absence of Dox, miRNAs were expressed at levels resembling that of the NC, which had no primary miRNA sequence inserted. However, in the presence of Dox, these three miRNAs were expressed at 7.4-, 27.0- and 45.5-fold higher levels than in the case without Dox induction, respectively (Fig. 3b). To precisely regulate miRNA expression, we induced miR-210-3p and miR-21-5p under different Dox concentrations. The levels of both miRNAs were gradually enhanced with increasing Dox, demonstrating a dose-dependent effect of the system in miRNA expression (Fig. 3c). Interestingly, a Dox concentration over 1,000 ng/mL had a minute inhibitory impact on miRNA expression since the miRNA level slightly decreased at 2,000–4,000 ng/mL. The concomitant expression of the EGFP upstream of primary miRNAs was visualized under a fluorescence microscope, which also manifested a Dox dose-dependent effect (Additional file 1).

In a time-dependent study, the level of miR-210-3p and miR-21-5p gradually increased within 2 days (Fig. 3d). However, after Dox withdrawal, the miRNA levels gradually decreased and resembled those before induction during the following 4 days, suggesting that the Tet-on system has a stringent time-dependent and reversible effect on miRNA inducibility.

Programmed cell death protein 4 (PDCD4) is a direct target of miR-21-5p [23]. It was interesting to test whether PDCD4 displayed quantitative regulation by miR-21-5p with the Tet-on system. Western blot analysis showed that the level of PDCD4 protein gradually decreased as the Dox level increased and reached 80% downregulation at 500 ng/mL of Dox (Fig. 3e and f). These results indicated that the optimized Tet-on system allowed a precise and convenient regulation of the expression of miRNA and its target.

### Tet-on control of CRISPR/Cas9 genome editing

**Fig. 4:**
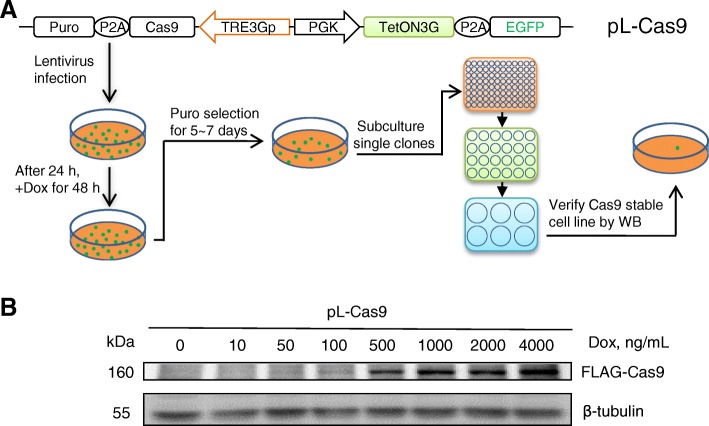
Development of an inducible Cas9 stable cell line. **a** HEK293A cells infected with lentiviruses carrying inducible FLAG-Cas9 cassette were treated with Dox for 48 h and then selected with puromycin for 5–7 d. Single cells were picked up and cultured sequentially in 96-, 24-, and 6-well plates with puromycin for several days. Positive Cas9 cell line was determined by anti-FLAG tag western blotting analysis. **b** The FLAG-Cas9 protein levels of the stable cell line under different Dox concentrations were assayed by western blotting. Beta-tubulin served as a loading control

CRISPR/Cas9 is a powerful approach for genome editing. However, upon continuous expression of Cas9, there is potentially a high risk of off-target effects [24–26]. In this study, we used the optimized Tet-on vector to construct an inducible Cas9 system, pL-Cas9. We first attempted to develop a Cas9 stably expressing cell line (Fig. 4a). It is of high efficiency screening the positive Cas9 cell lines through the puromycin resistance gene co-expressing with Cas9. We successfully obtained six Cas9 stable cell lines from seven clones picked. These six cell lines displayed similar expression of Cas9 (data not shown). We randomly chose one of them to perform a gradual induction with Dox. Western blot analysis showed a weak band of Cas9 protein at 50 ng/mL Dox, and its level was gradually enhanced with the addition of Dox, reaching significant expression among 500–4,000 ng/mL Dox (Fig. 4b).

**Fig. 5:**
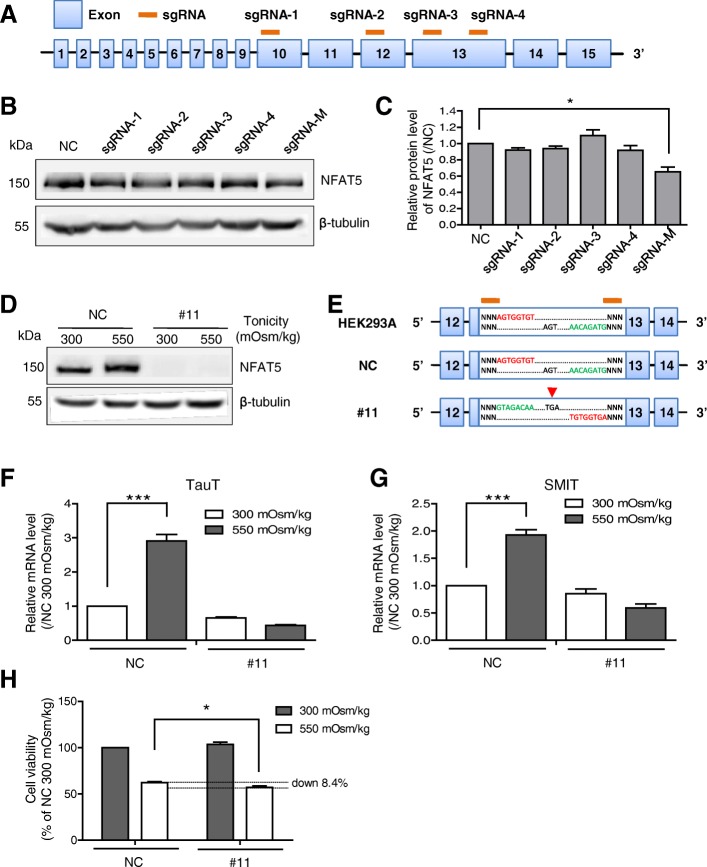
Knocking out the *NFAT5* gene with the inducible Cas9 Tet-on system. **a** Schematic representation of sgRNAs targeting the exonic region of the *NFAT5* gene. **b** Western blot analysis of *NFAT5* in Cas9 stable cells transfected with a single sgRNA (sgRNA-1, - 2, - 3, and - 4) or a mixture of them (sgRNA-M). Beta-tubulin served as a loading control. **c** The *NFAT5* protein levels were quantified. The data are expressed relative to the NC group transfected with an sgRNA targeting the *RFP*. *P < 0.05. **d** *NFAT5* expression in the knockout cell line. The NC transfected with sgRNA that targets the *RFP* and the #11 cell line transfected with sgRNA that targets *NFAT5* were cultured in isotonic (300 mOsm/kg) or hypertonic (550 mOsm/kg) media for 8 h. **e** Schematic representation of *NFAT5* gene editing in wild-type HEK293A, NC, and #11 cells. A ~ 310-nt fragment in exon 13 between the sgRNA-3 and sgRNA-4 recognition sites was inverted in #11. The premature stop codon TGA is indicated by a red arrow. **f, g** TauT and SMIT mRNA levels in NC and #11 cell lines under isotonic or hypertonic conditions for 8 h were measured by qRT-PCR with β-actin as an internal control. Bar charts show the relative expression level by normalization to the level of the NC group under isotonicity. Data are presented as mean ± SD (*n* = 3). ***P < 0.001. **h** Impact of *NFAT5* disruption on cellular viability under hypertonicity. The NC and #11 cell lines were cultured in isotonic or hypertonic media for 24 h and subjected to a cell viability assay by the MTS method. Bar charts show the relative viability by normalization to the level of the NC group under isotonicity. Data are presented as mean ± SD (*n* = 6). *P < 0.05

Next, we chose *NFAT5*, a protective transcription factor under osmotic stress, as a target in the following knockout study. Using an online tool, Broad Institute CRISPRko, four sgRNAs were designed and synthesized *in vitro*. Among them, sgRNA-1 targets exon 10, sgRNA-2 targets exon 12, and both sgRNA-3 and sgRNA-4 target exon 13 of the *NFAT5* gene (Fig. 5a, Additional file 3).

We then transfected the Cas9 stable cells with individual sgRNA or a mixture of them. To obtain an overall estimate of the mutation efficiency, a T7 endonuclease I (T7E1) assay was performed. The percentage of cleavage was between 6~17% (Additional file 2). Then, we evaluated the *NFAT5* protein level in the pooled cell samples. Western blot analysis showed that, despite being visible in each samples tested, the *NFAT5* protein exhibited a more significant reduction in the cells transfected with the mixture of sgRNAs (~ 35% decrease) than in those transfected with a single sgRNA (most with a ~ 10% decrease) (Fig. 5b and c).

We hence used the cells transfected with the mixed sgRNAs as the starting material to pick up and screen the *NFAT5*-knockout clones. From 14 screened clones, we successfully obtained an *NFAT5*-defective cell line, #11. As Fig. 5d showed, in the NC transfected with an sgRNA targeting the *RFP*, modest and upregulated expression of *NFAT5* was observed under isotonic (300 mOsm/kg) and hypertonic (550 mOsm/kg) conditions, respectively, which was consistent with that hypertonicity induces *NFAT5* expression [27]. However, *NFAT5* protein was completely lost in #11 cell line (Fig. 5d) under both osmotic conditions, suggesting that the *NFAT5* expression cassette in #11 cell line was damaged. We performed PCR and then DNA sequencing analysis of the *NFAT5* coding sequence of the cell line. It showed that a ~ 310-nucleotide (nt) fragment between the sgRNA-3 and sgRNA-4 recognition sites in exon 13 had been inverted in its original locus, suggesting an occurrence of double cleavage guided by sgRNA-3 and sgRNA-4. It possibly explained one of the reasons why the *NFAT5* protein exhibited a more significant reduction in the cells transfected with the mixture of sgRNAs than in those transfected with a single sgRNA in Fig. 5c. Such an inversion of exon fragment leads to a premature translation termination of the *NFAT5* protein, which might be unstable and susceptible to degradation (Fig. 5e, Additional file 3).

Taurine transporter (TauT) and sodium *myo*-inositol transporter (SMIT) are two important downstream targets of *NFAT5* [28]. We tested their mRNA levels under isotonic and hypertonic conditions by qRT-PCR. As shown in Fig. 5f and g, TauT and SMIT in the NC group were increased 2.9- and 1.9-fold under hypertonic conditions compared with their levels under isotonic ones. However, neither TauT nor SMIT in #11 could be activated under hypertonic condition, confirming that the *NFAT5* gene was disrupted in the chromosome. Given that *NFAT5* has a protective function under osmotic stress, we were interested in whether the deficiency of *NFAT5* gene would influence the ability of cells to resist hypertonicity. The MTS cell viability assay revealed that knocking out *NFAT5* led to a 9% decrease of cell viability compared with that in the NC group under hypertonic condition, confirming the importance of *NFAT5* in osmoregulation (Fig. 5h).

## Discussion

Tetracycline inducible system (Tet-on and Tet-off) has been extensively applied in biological study *in vitro* and *in vivo* due to it having a number of advantages, including inducibility, a broad dynamic range, and reversibility. This study aimed to improve the Tet-on system with superior regulatory characteristics. By systematically comparing the two transactivators TetON3G and rtTA3; the three TREs TRE3Gp, TRE3Gs, and TetO6; both orientations of induced expression cassette; and the five promoters of P_PGK_, P_CMV_, P_EF1α_, P_SV40_, and P_Ubc_ controlling transactivator expression, we successfully identified that the design of the lentiviral p2 construct is the best Tet-on system tested.

Between the two rtTAs studied, TetON3G displayed higher transcriptional capacity than rtTA3 did. Both TetON3G and rtTA3 were fusions of TetR and VP16-derived minimal activation domain (AD). In contrast to two minimal ADs contained in rtTA3, three ADs were harbored in TetON3G, which possibly enabled the higher transactivation ability [29] (Fig. 1a). Apart from the difference of AD numbers, there were three amino acids inconsistent between TetON3G and rtTA3 at the positions 12, 67, and 171. They were S, S, and K in TetON3G, but G, F, and R in rtTA3, respectively. As reported previously, S12G increased the sensitivity to Dox [4], while F67S and R171K conferred both enhanced transcriptional activity and Dox sensitivity [7]. It would be interesting to test whether better results can be acquired when S12G mutation is also introduced in TetON3G.

The TREs are of great importance for the inducibility. TRE3Gs and TetO6 are frequently used TREs. Moreover, TRE3Gp, a modified type of TRE3G originating from the plasmid pLVX-TRE3G-ZsGreen1 (Clontech) with a truncated CMV minimal promoter, also has great potential [30]. In contrast to containing six TetO1s in TetO6, both TRE3Gs and TRE3Gp possess seven TetOs, possibly allowing higher affinity to rtTA. The differences among these three TREs were also located in the 5′-UTR sequence of the CMV minimal promoter (Fig. 1b, Additional file 3). To reduce the background expression, a 5′-UTR fragment of Turnip Yellow Mosaic virus was fused downstream of the initiator sequence of CMV minimal promoter in both TRE3Gs and TRE3Gp, which might prevent recognition by other transcription factors in mammalian cells [9]. With a shorter 5′-UTR sequence, TRE3Gp possesses increased sensitivity to Dox and fold induction when compared with TRE3Gs. We thus selected the TRE3Gp-containing system, namely, p2 plasmid, in the subsequent application study. As the basal expression level of TRE3Gp was a little higher than TRE3Gs, p1 might be better when leakiness or toxicity of the gene is the major concern.

The promoter controlling the transactivator production is another critical factor influencing the efficacy of Tet-on system. As previously reported, P_CMV_, P_EF1α_, and P_SV40_ displayed strong efficiency in controlling gene expression, while P_PGK_ revealed a relatively weak level [31]. However, the fold induction of P_PGK_ was the optimal among them. Promoter activities are believed to be conserved within mammalian tissues [31, 32], which was confirmed in this study that P_PGK_ is consistently dominant in multiple human and mouse cell lines. CMV promoter was an exception in that it showed variability from one cell type to another. Such variation might be due to different levels of promoter silencing by DNA methylation in different cell types [33–35].

We positioned the induced expression cassette in two orientations. The results showed that the TRE3Gs expression cassettes in the same orientation displayed higher leaky activity, indicating an orientation-dependent effect of TRE3Gs. Positioning the expression cassettes in the same orientation probably led to transcriptional read-through by the lentiviral 5′ LTR promoter [36]. In this case, the induced gene could be activated without Dox induction when the transactivator sequence positioned in the same orientation was constitutively transcribed and read through. However, since no significant leaky background activities were found with TRE3Gp and TetO6, such a read-through effect seemed to be TRE3Gs-dependent.

miRNA can post-transcriptionally regulate gene expression via binding to the 3′-UTR sequence of its target mRNA. As demonstrated in this paper, a gradual increase of Dox induced gradual elevation of the miR-21-5p level, which then caused a gradual reduction of PDCD4 protein, with the lowest at 500 ng/mL of Dox. PDCD4 level slightly increased at 1,000 and 2,000 ng/mL of Dox, seemingly concordant with the slight inhibition of miR-21-5p expression at high levels of Dox (Fig. 3e and f). This constitutes more solid evidence that PDCD4 is a direct target of miR-21-5p. In the conventional miRNA studies, synthetic miRNA mimic were used to transfect into the target cells. The concentration of miRNA mimic in cells, however, might be inconsistent between batches due to the variation of transfection efficiency. The optimized Tet-on system can easily accomplish a precise, gradient expression of miRNA, which is also in low cost by adding different amount of Dox. Overall, the optimized Tet-on system provides a more cost-effective and precise fine-tuning to confirm the target gene of a miRNA.

CRISPR/Cas9 is a rapid and efficient approach for genetic perturbation [37, 38]. In this study, we developed a simplified strategy facilitating the Cas9 application. By infection with an all-in-one lentivirus harboring rtTA and Cas9 expression cassettes, a cell line stably expressing inducible Cas9 soon became available. The usage of a short 2A peptide greatly reduces the size of the lentiviral vector and improves the viral titer. With the stable cell line, we can conveniently perform CRISPR/Cas9 experiments merely by introducing the *in vitro*-transcribed sgRNA, which is small (~ 100 nt) and easily transfected into cells. This system offers great convenience in sgRNAs library screening. By contrast, the protocol from Clontech is relatively laborious and liable to disturb cell viability, which recommends the sequential transduction of three different lentiviruses encoding TetON3G, Cas9, and sgRNA (https://www.takarabio.com; Cat. No. 632633, published on December 15, 2016).

## Conclusions

This work explored a series of combinations of Tet-on components and identified an optimal configuration for effective and stringent gene regulation for genetic investigations in cellular and animal sciences.

## Additional files

Additional file 1: **Figure S1.** Concomitant expression of EGFP under different dox levels. EGFP and the downstream primary miRNA sequence were positioned under the control of the TREs. miRNA and EGFP were induced under different concentrations of Dox (0, 10, 50, 100, 500, 1,000, 2,000, and 4,000 ng/mL) for 48 h. The expression of the EGFP was examined under a fluorescence microscope. (PDF 101 kb)

Additional file 2: **Figure S2.** Targeted mutations revealed by T7E1 assay. The PCR products from genomic DNA of cells transfected with sgRNA targeting *RFP* (NC) and *NFAT5* were treated (+) or untreated (**−**) with T7E1 after melting and annealing. Arrows indicate the cleaved fragments by T7E1. The mutation efficiency is shown at the bottom. (PDF 89 kb)

Additional file 3: Supplementary data. Primers, probe, DNA sequences, and sgRNA information. (PDF 178 kb)

## Abbreviations

AD: Activation domain
BD: Binding domain
Dox: Doxycycline
EGFP: Enhanced green fluorescent protein
Inr: Initiator
Luc: Luciferase
miRNAs: MicroRNAs
NC: Negative control
NFAT5: Nuclear factor of activated T cells-5
nt: Nucleotide
PDCD4: Programmed cell death protein 4
PEI: Polyethylenimine
*RFP*: Red fluorescence protein
rtTA: Reverse tTA
rtTA3: Tetracycline-transactivator 3
SD: Standard deviation
SMIT: Sodium myo-inositol transporter
TauT: Taurine transporter
Tet: Tetracycline
TetOs: Tet operators
TetR: Tet repressor
TREs: Tet-responsive elements
tTA: Tet-regulated transactivator
