# Supplementary material for: An improved Tet-on system in microRNA overexpression and CRISPR/Cas9-mediated gene editing
Source: J Anim Sci Biotechnol. 2019 Jun 10;10:43. doi: 10.1186/s40104-019-0354-5 (PMC6556963; doi:10.1186/s40104-019-0354-5)
Supplement: Supplementary file 1 — Figure S1. Concomitant expression of EGFP under different dox levels. EGFP and the downstream primary miRNA sequence were positioned under the control of the TREs. miRNA and EGFP were induced under different concentrations of Dox (0, 10, 50, 100, 500, 1,000, 2,000, and 4,000 ng/mL) for 48 h. The expression of the EGFP was examined under a fluorescence microscope. (PDF 101 kb) [file 40104_2019_354_MOESM1_ESM.pdf]

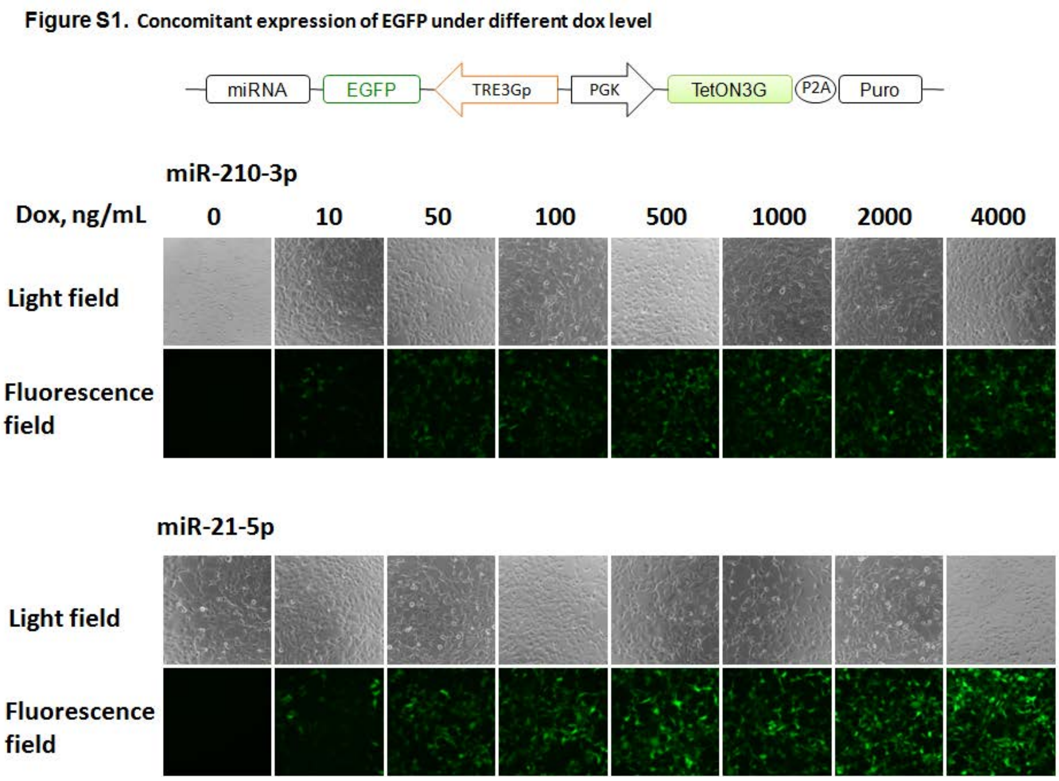

Figure S1. Concomitant expression of EGFP under different dox level. The EGFP and the downstream primary miRNA sequence were positioned under the control of the TREs. miRNA and EGFP were induced under different concentrations of Dox (0, 10, 50, 100, 500, 1000, 2000, and 4000 ng/ml) for 48 h. The expression of the EGFP was examined under a fluorescence microscope.
