# Supplementary material for: An improved Tet-on system in microRNA overexpression and CRISPR/Cas9-mediated gene editing
Source: J Anim Sci Biotechnol. 2019 Jun 10;10:43. doi: 10.1186/s40104-019-0354-5 (PMC6556963; doi:10.1186/s40104-019-0354-5)
Supplement: Supplementary file 2 — Figure S2. Targeted mutations revealed by T7E1 assay. The PCR products from genomic DNA of cells transfected with sgRNA targeting RFP (NC) and NFAT5 were treated (+) or untreated (−) with T7E1 after melting and annealing. Arrows indicate the cleaved fragments by T7E1. The mutation efficiency is shown at the bottom. (PDF 89 kb) [file 40104_2019_354_MOESM2_ESM.pdf]

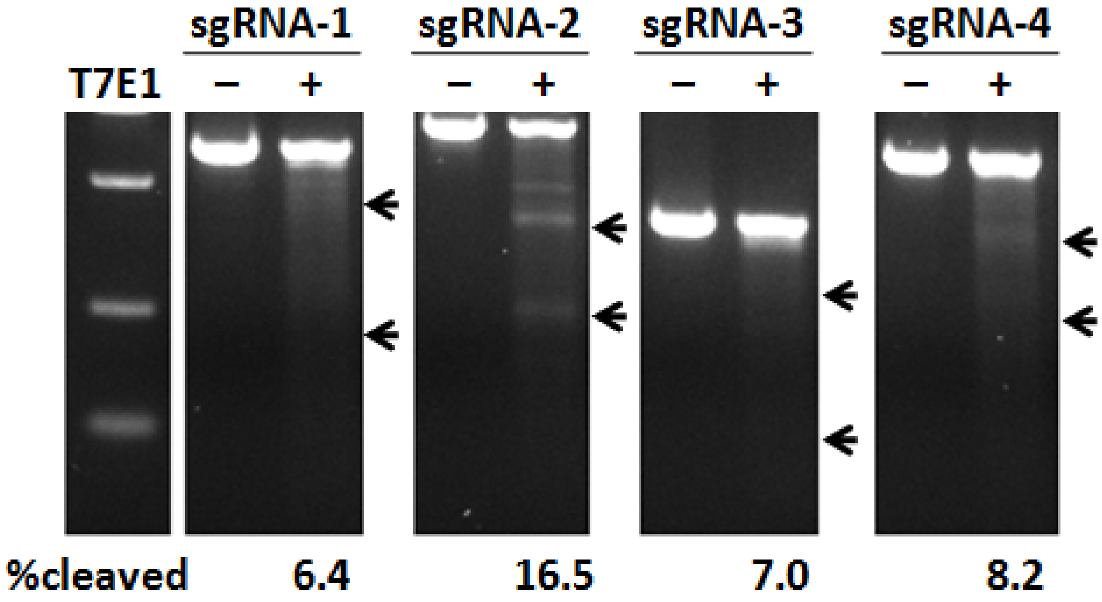

Figure S2. Targeted mutations revealed by T7E1 assay. The DNA fragment PCR-amplified around the CRISPR/Cas9 target sites from genomic DNA of cells transfected with sgRNA targeting either *rfp* (NC) or *Nfat5* was treated (+) or untreated (**–**) with T7E1. Arrows indicate the cleaved fragments by T7E1. The mutation efficiency is shown at the bottom.
