# Supplementary material for: An improved Tet-on system in microRNA overexpression and CRISPR/Cas9-mediated gene editing
Source: J Anim Sci Biotechnol. 2019 Jun 10;10:43. doi: 10.1186/s40104-019-0354-5 (PMC6556963; doi:10.1186/s40104-019-0354-5)
Supplement: Supplementary file 3 — Supplementary data. Primers, probe, DNA sequences, and sgRNA information. (PDF 178 kb) [file 40104_2019_354_MOESM3_ESM.pdf]

Additional file 3: Supplementary data. Primers, probe, DNA sequences and sgRNA information.

Table 1. Primers and probe

miRNA/Gene	Primer	Sequence (5'-3')	Details
>hsa-miR-210-3p MIMAT0000267 CUGUGCGUGUGACA GCGGCUGA	RT primer	CAGTGCAGGGTCCGAGGTCAGAGCCACCTGGG CAATTTTTTTTTTTCAGCCG	miRNA qRT-PCR
	Forward primer	GGCTGTGCGTGTGACAGC	
>hsa-miR-21-5p MIMAT0000076 UAGCUUAUCAGACU GAUGUUGA	RT primer	CAGTGCAGGGTCCGAGGTCAGAGCCACCTGGG CAATTTTTTTTTTTCAACAT	
	Forward primer	CTCGGTAGCTTATCAGACTG	
>hsa-miR-26a-5p MIMAT0000082 UUCAAGUAAUCCAG GAUAGGCU	RT primer	GTGCAGGGTCCGAGGTCAGAGCCACCTGGGCA ATTTTTTTTTTTAGCCTA	
	Forward primer	CCGGGTTCAAGTAATCCAGGA	
SNORD44 NR_002750.2	RT primer	GTGCAGGGTCCGAGGTCAGAGCCACCTGGGCA ATTTTTTTTTTTAGTCAG	
	Forward primer	CATGAAGGTCTTAATTAGCTCTA	
	Universal reverse primer	CAGTGCAGGGTCCGAGGT	
	Universal Taqman probe	56-FAM/CAGAGCCAC/ZEN/CTGGGCAATTT/3I ABkFQ	
hNFAT5	hNFAT5 F1	CTCCTCAGATTCAGTTGGTACA	mRNA qRT-PCR
	hNFAT5 R1	GACTGTTGCATATCTTGTTGAT	
hTauT	hTauT-F	AGATCATCATAGGCCAGTACAC	
	hTauT-R	TAGACATTCAGGAGGGACACA	
hSMIT	hSMIT-F	GGCAGCCTATCTCTTCCATATC	
	hSMIT-R	GAGGGACCCTAATTCTCCAAAC	
β-actin	β-actin-F	AAAGACCTGTACGCCAACAC	
	β-actin-R	GTCATACTCCTGCTTGCTGAT	
NFAT5	T7-sg-1-F	TTCTAATACGACTCACTATAGCAAGTCAGTCAAA TTCAGAGGTTTTAGAGCTAGA	sgRNA *in vitro* synthesis
	T7-sg-2-F	TTCTAATACGACTCACTATAGTGGTATGAGTGG AGTCATCAGTTTTAGAGCTAGA	
	T7-sg-3-F	TTCTAATACGACTCACTATAGAGTGCATTGTCTA CCAATGGTTTTAGAGCTAGA	
	T7-sg-4-F	TTCTAATACGACTCACTATAGATATGTTGGTCAT GATAGGGGTTTTAGAGCTAGA	
RFP	T7-sg-NC-F	TTCTAATACGACTCACTATAGCCACAGACTGGA AAGAATCAGTTTTAGAGCTAGA	
	Universal-sgRNA-R	AAAAAGCACCGACTCGGTGCCACTT	

MCS-PPGK-TetON3G-P2A-Puro	Tet2A-Puro-F	CATGATCGATACGCGTGAATTCCTCGAG	
	Tet2A-Puro-R	ACTAGTTCAGGCACCGGGCTTGCGGGT	
Luc	Luc-F	AAGAATGCGGCCGCATGGAAGATGCCAAAAACATTAAG	
	Luc-R	GATGACGCGT*CGTCTCTCTAGA*TTACACGGCGATCTTGCCGCCCTT	
rtTA3	rtTA3-F	TGCATTCGAAATGTCTAGGCTGGACAAGAGCA	
	rtTA3-R	ATATCCCGGGGAGCATGTCAAGGTCA	
P_{CMV}	CMV-F	CATGTCTAGATATTAATAGTAATCAATTACG	
	CMV-R	TGCATTCGAAGCCAGTAAGCAGTGGGTTCTC	
$P_{EF1\alpha}$	EF1α-F	CATGTCTAGAAAGGATCTGCGATCGCTCCG	
	EF1α-R	TGCATTCGAACACAGCTTGGATCTGTAACG	
P_{SV40}	SV40-F	CATGTCTAGAGCGCAGCACCATGGCCTGA	
	SV40-R	TGCATTCGAAAAGCTTTTTGCAAAAGCCTAG	
P_{ubc}	Ubc-F	CATGTCTAGAGGCCTCCGCGCCGGGTTTTG	
	Ubc-R	TGCATTCGAAATGGATCCGCTAGCGTCTAAC	
EGFP	EGFP-F1	AAGAATGCGGCCGCATGGTGAGCAAGGGCGA	miRNA vector
	EGFP-R1	GATGACGCGTTTACTTGTACAGCTCGTCC	
EGFP	EGFP-F2	CATCGGATCCATGGTGAGCAAGGGCGA	cas9 vector
	EGFP-R2	CATGACTAGTTTACTTGTACAGCTCGTCC	
pri-hsa-miR-210	miR-210-F	GATGACGCGTCTGAAGTTGGGCCGAGAG	Primary miRNA
	miR-210-R	CATGATCGATGTATCTGGCCCAGCCTCA	
pri-hsa-miR-21	miR-21-F	GATGACGCGTCCTTTAGGAGCATTATGAGC	
	miR-21-R	CATGATCGATATCCTCCCTCCATACTGCTG	
pri-hsa-miR-16-2	miR-16-F	GATGACGCGTCCTTAAAGTACTGTAGCAGCACAT	
	miR-16-R	CATGATCGATGGTAAATCAAACACCAAGTGTACAG	
pri-hsa-miR-26a-2	miR-26a-F	GATGACGCGTCAGAAGCAAGCACAAGATCGG	
	miR-26a-R	CATGATCGATACCAGTTACACAGCAGCAAGG	
pri-hsa-miR-32	miR-32-F	GATGACGCGTCACTATCTCATTGAAGTTTTG	
	miR-32-R	CATGATCGATGATTTACTAAATGATCATTGC	
Cas9-FLAG-P2A-Puro	Cas9-2A-puro-F	AAGAATGCGGCCGCATGGACAAGAAGTACAGCATCG	
	Cas9-2A-puro-R	GATGACGCGTTCAGGCACCGGGCTTGCGGGT	
NFAT5	T7E1-sg-1-F	ATCCTGATGCCTGTGAACCTGAGCT	T7E1 assay
	T7E1-sg-1-R	GCACATAAGCGGTAGTCAATGCATGG	
	T7E1-sg-2-F	TTCCAGAGGTCCCCCAGCCAATGCA	
	T7E1-sg-2-R	GGCTCCTCAGTAACATTACAGGGGATAAGA	
	T7E1-sg-3-F	CCAGATGGTAATGAGAATGTTCAAG	
	T7E1-sg-3-R	GGAGAAGAAAACAATTCACACTGCA	
	T7E1-sg-4-F	CCAGATGGTAATGAGAATGTTCAAG	
	T7E1-sg-4-R	GAGGTTGATTGTCCCCACTATGTTGCA	

Luciferase	Tet-5956-luc F1	TACACCTTCGTGACTTCCCATTTGC	qPCR
	Tet-5956-luc R1	GCATGACTGAATCGGACACAAG	

Sequence of artificially synthesized DNA

Note: Tet operators are underlined. TFIIB is shaded in grey. TATA box is indicated in red. Predicted initiator (Inr) is shaded in grey and underlined. Restriction sites are displayed in lower case.

(1) TRE3Gs

tctagacgtctcacgcgtTTTACTCCCTATCAGTGATAGAGAACGTATGAAGAGTTTACTCCCTATCAGTGATAGAGA
ACGTATGCAGACTTTACTCCCTATCAGTGATAGAGAACGTATAAGGAGTTTACTCCCTATCAGTGATAGAGAA
CGTATGACCAGTTTACTCCCTATCAGTGATAGAGAACGTATCTACAGTTTACTCCCTATCAGTGATAGAGAACG
TATATCCAGTTTACTCCCTATCAGTGATAGAGAACGTATAAGCTTTGCTTATGTAAACCAGGGCGCCTATAAAA
GAGTGCTGATTTTTTGAGTAAACTTCAATTCCACAACACTTTTGTCTTATACCAACTTTCCGTACCACTTCCTA
CCCTCGTAAAgcggccgc

(2) TRE3Gp

tctagacgtctcacgcgtTTTACTCCCTATCAGTGATAGAGAACGTATGAAGAGTTTACTCCCTATCAGTGATAGAGA
ACGTATGCAGACTTTACTCCCTATCAGTGATAGAGAACGTATAAGGAGTTTACTCCCTATCAGTGATAGAGAA
CGTATGACCAGTTTACTCCCTATCAGTGATAGAGAACGTATCTACAGTTTACTCCCTATCAGTGATAGAGAACG
TATATCCAGTTTACTCCCTATCAGTGATAGAGAACGTATAAGCTTTAGGCGTGTACGGTGGGCGCCTATAAAA
GCAGAGCTCGTTTAGTGAACCGTCAGATCGCCTGGAGCAATTCCACAACACTTTTGTCTTATACgcggccgc

(3) TetO6

tctagacgtctcacgcgtTTTACTCCCTATCAGTGATAGAGAACGATGTCGAGTTTACTCCCTATCAGTGATAGAGAA
CGTATGTCGAGTTTACTCCCTATCAGTGATAGAGAACGTATGTCGAGTTTACTCCCTATCAGTGATAGAGAAC
GTATGTCGAGTTTATCCCTATCAGTGATAGAGAACGTATGTCGAGTTTACTCCCTATCAGTGATAGAGAACGTA
TGTCGAGGTAGGCGTGTACGGTGGGAGGCCTATATAAGCAGAGCTCGTTTAGTGAACCGTCAGATCGCgcgg
ccgc

(4) MCS-P_{PGK}-TetON3G-P2A-Puro

atcgatacgcgtGAATTCCTCGAGACCGGTgcggccgctctagaGGGGTTGGGGTTGCGCCTTTTCCAAGGCAGCCC
TGGGTTTGCGCAGGGACGCGGCTGCTCTGGGCGTGGTTCCGGGAAACGCAGCGGCGCCGACCCTGGGTC
TCGCACATTCTTCACGTCCGTTCGCAGCGTCACCCGGATCTTCGCCGCTACCCTTGTGGGCCCCCCGGCGAC
GCTTCCTGCTCCGCCCCTAAGTCGGGAAGGTTCCTTGCGGTTCGCGGCGTGCCGGACGTGACAAACGGAA
GCCGCACGTCTACCTAGTACCCTCGCAGACGGACAGCGCCAGGGAGCAATGGCAGCGCGCCGACCGCGAT
GGGCTGTGGCCAATAGCGGCTGCTCAGCAGGGCGCGCCGAGAGCAGCGGCCGGGAAGGGGCGGTGCGG
GAGGCGGGGTGTGGGGCGGTAGTGTGGGCCCTGTTCCTGCCCGCGCGGTGTTCCGCATTCTGCAAGCCTC
CGGAGCGCACGTCGGCAGTCGGCTCCCTCGTTGACCGAATCACCGACCTCTCTCCCCAGGGttcgaaCACCAT
GTCAAGGCTGGACAAGAGCAAAGTCATAAACTCTGCTCTGGAATTACTCAATGGAGTCGGTATCGAAGGCCT
GACGACAAGGAAACTCGCTCAAAAGCTGGGAGTTGAGCAGCCTACCCTGTACTGGCACGTGAAGAACAAG
CGGGCCCTGCTCGATGCCCTGCCAATCGAGATGCTGGACAGGCATCATACCCACTCCTGCCCCCTGGAAGGC
GAGTCATGGCAAGACTTTCTGCGGAACAACGCCAAGTCATACCGCTGTGCTCTCCTCTCACATCGCGACGGG
GCTAAAGTGCATCTCGGCACCCGCCCAACAGAGAAACAGTACGAAACCCTGGAAAATCAGCTCGCGTTCCT

GTGTCAGCAAGGCTTCTCCCTGGAGAACGCACTGTACGCTCTGTCCGCCGTGGGCCACTTTACACTGGGCT
GCGTATTGGAGGAACAGGAGCATCAAGTAGCAAAAGAGGAAAGAGAGACACCTACCACCGATTCTATGCCC
CCACTTCTGAAACAAGCAATTGAGCTGTTCGACCGGCAGGGAGCCGAACCTGCCTTCCTTTTCGGCCTGGA
ACTAATCATATGTGGCCTGGAGAAACAGCTAAAGTGCGAAAGCGGCGGGCCGACCGACGCCCTTGACGATT
TTGACTTAGACATGCTCCCAGCCGATGCCCTTGACGACTTTGACCTTGATATGCTGCCTGCTGACGCTCTTGA
CGATTTTGACCTTGACATGCTCcccgggAGCGGAGCTACTAACTTCAGCCTGCTGAAGCAGGCTGGCGACGT
GGAGGAGAACCCTGGACCTggatccATGACCGAGTACAAGCCCACGGTGCGCCTCGCCACCCGCGACGACGT
CCCCAGGGCCGTACGCACCCTCGCCGCCGCGTTCGCCGACTACCCCGCCACGCGCCACACCGTCGATCCGG
ACCGCCACATCGAGCGGGTCACCGAGCTGCAAGAACTCTTCCTCACGCGCGTCGGGCTCGACATCGGCAA
GGTGTGGGTCGCGGACGACGGCGCCGCCGTGGCGGTCTGGACCACGCCGGAGAGCGTCGAAGCGGGGG
CGGTGTTCGCCGAGATCGGCCCGCGCATGGCCGAGTTGAGCGGTTCCCGGCTGGCCGCGCAGCAACAGAT
GGAAGGCCTCCTGGCGCCGCACCGGCCCAAGGAGCCCGCGTGGTTCCTGGCCACCGTCGGCGTGTCGCCC
GACCACCAGGGCAAGGGTCTGGGCAGCGCCGTCGTGCTCCCCGGAGTGGAGGCGGCCGAGCGCGCCGG
GGTGCCCGCCTTCCTGGAGACCTCCGCGCCCCGCAACCTCCCCTTCTACGAGCGGCTCGGCTTCACCGTCAC
CGCCGACGTCGAGGTGCCCGAAGGACCGCGCACCTGGTGCATGACCCGCAAGCCCGGTGCCTGAactagt

Table 2. Information of sgRNA target sequences

Target Gene	Target Transcript	Strand	sgRNA Target Sequence (5'-3')	PAM Sequence	Exon Number
NFAT5	NM_138713.3	antisense	ATATGTTGGTCATGATAGGG	AGG	10
		antisense	TGGTATGAGTGGAGTCATCA	GGG	12
		sense	CAAGTCAGTCAAATTCAGAG	TGG	13
		sense	GAGTGCATTGTCTACCAATG	AGG	13
RFP	KT358727.1	sense	CCACAGACTGGAAAGAATCA	AGG	

DNA sequencing of *nfat5* PCR products

Note: sgRNA targeting sequences are underlined. PAM sequences are highlighted in bold italic. The predicted cutting sites of Cas9 are marked in red. The premature termination codon TGA in #11 *nfat5* exon 13 was marked in bold, red font.

(1) HEK-293A *nfat5* exon 13

…

TTTTTCAACAAGTCAGTCAAATTCAGAG***TGG***TGTAAGCCCTGGAATGTTTTCCTCAACAGAGCCAACAGTCCATAC
CAGACCAGATAATTTATTACCTGGAAGAGCTGAAAGTGTTCATCCACAGTCTGAAAACACGTTATCTAATCAACAG
CAGCAGCAGCAGCAGCAACAGCAAGTGATGGAATCTTCAGCCGCAATGGTGATGGAGATGCAACAGAGTATCTG
CCAGGCAGCTGCCCAGATTCAGTCAGAGTTATTCCCTTCAACTGCTTCAGCAAATGGAAACCTTCAGCAATCGCCA
GTTTACCAGCAGACTTCTCACATGATGAGTGCATTGTCTACCAATG***AGG***ATATGCAAATGCAGTGTGAATTG…

(2) NC *nfat5* exon 13

…

TTTTTCAACAAGTCAGTCAAATTCAGAG***TGG***TGTAAGCCCTGGAATGTTTTCCTCAACAGAGCCAACAGTCCATAC

CAGACCAGATAATTTATTACCTGGAAGAGCTGAAAGTGTTCATCCACAGTCTGAAAACACGTTATCTAATCAACAG

CAGCAGCAGCAGCAGCAACAGCAAGTGATGGAATCTTCAGCCGCAATGGTGATGGAGATGCAACAGAGTATCTG

CCAGGCAGCTGCCCAGATTCAGTCAGAGTTATTCCCTTCAACTGCTTCAGCAAATGGAAACCTTCAGCAATCGCCA

GTTTACCAGCAGACTTCTCACATGATGAGTGCATTGTCTACCAATG***AGG***ATATGCAAATGCAGTGTGAATTG…

(3) #11 *nfat5* exon 13

…

TTTTTCAACCAAGTCAGTCAAATTCAG**TGA**GAAGTCTGCTGGTAAACTGGCGATTG

CTGAAGGTTTCCATTTGCTGAAGCAGTTGAAGGGAATAACTCTGACTGAATCTGGGCAGCTGCCTGGCAGATACT

CTGTTGCATCTCCATCACCATTGCGGCTGAAGATTCCATCACTTGCTGTTGCTGCTGCTGCTGCTGTTGATTAG

ATAACGTGTTTTCAGACTGTGGATGAACACTTTCAGCTCTTCCAGGTAATAAATTATCTGGTCTGGTATGGACTGTT

GGCTCTATTGAGGAAAACATTCCAGGGCTTACCACCACTCTAATG***AGG***ATATGCAAATGCAGTGTGAATTG…
